# A Study to Investigate Ghanaian Radiography Students' Proficiency, Experiences, Confidence, and Knowledge Concerning Communication With Hearing‐Impaired Patients

**DOI:** 10.1002/jmrs.70082

**Published:** 2026-03-20

**Authors:** Seth Kwadjo Angmorterh, Mariella Mawunyo Amoussou‐Gohoungo, Jerry Korley, Adam Inusah, Olawale Ogundiran, Sonia Aboagye, Patience Nyamekye Agyemang, Nathaniel Awentiirin Angaag, Wise Tsivanyo, Octavia Nyamekye Adjoa Amoabeng, Godfred Adusei, Jennifer Bannerman‐Williams, Nii Korley Kortei, Riaan van de Venter

**Affiliations:** ^1^ Department of Medical Imaging, School of Allied Health Sciences University of Health and Allied Sciences (UHAS) Ho Ghana; ^2^ Department of Speech, Language & Hearing Sciences, School of Allied Health Sciences University of Health and Allied Sciences (UHAS) Ho Ghana; ^3^ Department of Nutrition and Dietetics, School of Allied Health Sciences University of Health and Allied Sciences (UHAS) Ho Ghana; ^4^ Department of Sports Nutrition, School of Sports and Exercise Medicine University of Health and Allied Sciences (UHAS) Ho Ghana; ^5^ Department of Radiography, School of Clinical Care and Medicinal Sciences, Faculty of Health Sciences Nelson Mandela University Gqeberha South Africa

**Keywords:** communication, deaf, hearing, hearing disabilities, hearing‐impaired patients, radiography, students

## Abstract

**Introduction:**

Effective communication in healthcare improves patient outcomes, especially the hearing‐impaired. Ghanaian radiography students undertake clinical placements, rotating through units with different radiological modalities, and interacting with different patient groups, including patients with hearing disabilities. However, no study investigated their proficiency and experiences communicating with hearing‐impaired patients. This study aimed to investigate Ghanaian radiography students' proficiency, experiences, confidence, and knowledge concerning communication with hearing‐impaired patients.

**Methods:**

A quantitative approach with a descriptive cross‐sectional study design was employed. Convenience sampling was used to recruit undergraduate diagnostic radiography students from one Ghanaian public university. An online validated survey was used. Descriptive and inferential statistics were performed using the Statistical Package for Social Sciences (SPSS).

**Results:**

The study involved 152 participants [males = 93 (61.2%), females = 59 (38.8%)], aged 17–32 years (mean = 21 ± 2.3 years). Most participants could not communicate using sign language (*n* = 147, 96.7%). The majority of the participants had not interacted with hearing‐impaired patients during clinical placement (*n* = 103, 67.8%). Students reported both positive and negative experiences. There was a statistically significant association between year of study and students' interaction with hearing‐impaired patients (*p* = 0.017). Seventy‐six participants (50%) demonstrated low confidence in communicating effectively with hearing‐impaired patients. There was a statistically significant association between biological sex and students' confidence in communicating with hearing‐impaired patients (*p* = 0.043), with the male students expressing a higher confidence level.

**Conclusion:**

The majority of participants could not communicate using sign language, had no prior experience working with hearing‐impaired patients, and had low levels of confidence interacting with hearing‐impaired patients. Participants demonstrated some knowledge regarding care practices for hearing‐impaired patients. The study highlights a curriculum gap in preparing undergraduate diagnostic radiography students to provide effective care for hearing‐impaired patients.

## Graphical abstract

This study investigated undergraduate Ghanaian radiography students' proficiency, experiences, confidence, and knowledge with regard to communicating with hearing‐impaired patients. It was found that although most students had some knowledge about effective communication strategies to be used with hearing‐impaired patients, their confidence levels were low. It is recommended that students' knowledge and confidence be enhanced through appropriate learning and teaching strategies.

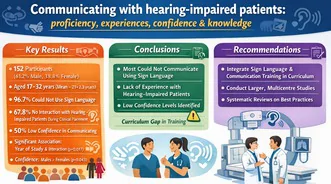

## 1 Introduction

Communication includes the exchange of information through verbal expressions and body language [1]. Effective communication between patients and healthcare professionals (HCPs), including radiographers, influences the overall quality of service provided because it improves healthcare coordination, patients' safety, quality of life and satisfaction, and decreases the cost of care by minimising errors and the need for repeats of radiographic examinations [2, 3, 4, 5, 6, 7, 8, 9, 10, 11, 12]. Similarly, effective communication strengthens patients' trust, promotes engagement, and enhances patients' adherence to treatment regimens [13, 14]. However, poor communication may result in safety incidents leading to mistrust, frustration, and fear, which could contribute to poorer patient health outcomes [15, 16]. Poor communication may also give rise to professional negligence and medical malpractice cases. The consequences of this can be the payment of huge sums of money as damages and the delicensing of HCPs [17, 18]. Additionally, patients with disabilities, such as the hearing‐impaired, may be impacted even more negatively by poor communication practices [19].

Hearing impairment is characterised by the loss or decreased ability to perceive sound, ranges from mild to severe, and may be unilateral or bilateral [20]. Globally, the burden of hearing impairment and its future projection is huge. For instance, approximately 466 million individuals are affected by hearing impairment, and this number is projected to increase to over 900 million by 2050 [21]. Specifically, the prevalence of hearing impairment across the United Kingdom (UK) and Germany is approximately 26 and 19%, respectively [22, 23]. In Africa, there are about 40 million hearing‐impaired people with varying statistics across countries [24]. For example, the prevalence of hearing impairment is 3.6% in Cameroon, 20% in South Africa, and 2% among the Ghanaian population [25, 26, 27]. According to Alshehri et al. [28] and Alamro et al. [29], many hearing‐impaired individuals have difficulty accessing healthcare facilities and understanding medical information, which could lead to misunderstandings concerning diagnosis, treatment plans, and instructions. Also, most HCPs are not prepared or willing to manage patients with hearing impairment [30]. This may be due to the incompetency of many HCPs in communicating with this patient group, and the absence of standard regulations for managing hearing‐impaired patients in most countries [9].

Several studies have been conducted to investigate the experiences of HCPs and/or interns in communicating with hearing‐impaired patients. A study by Alamro et al. [29] among Saudi Arabian HCPs showed that about 99% of them had poor knowledge and negative attitudes towards hearing‐impaired patients. Similar studies conducted in Namibia, Saudi Arabia, and South Africa concluded that HCPs found it difficult to communicate with hearing‐impaired patients because they could not use sign language [31, 32, 33]. Similarly, studies in Germany and the UK indicated poor attitudes and low levels of confidence among healthcare students in working with hearing‐impaired patients [23, 34]. Furthermore, a study by O'Riordan et al. [35] indicated the absence of comprehensive training of Irish radiography staff in communicating with hearing‐impaired patients. A similar study by Ogwudu and Akpaniwo [36] in the UK among radiographers showed distinct and diverse communication difficulties (e.g., the impact of accents, lip‐reading, inexperience, and working environment) while caring for hearing‐impaired patients. The study recommended that training of staff and technological solutions could help provide quality care for hearing‐impaired patients.

In contrast, a study conducted among radiography students in the UK indicated positive experiences towards communication with hearing‐impaired patients [9]. These experiences were influenced by year of study (clinical placement experience) and students' age. The research concluded that positive service adaptations such as lip‐reading, writing, the use of interpreters, speaking louder for hearing‐impaired patients, and the use of sign language were helpful in interactions with hearing‐impaired patients [9]. Radiography training and practice are well established in Ghana, and students periodically undertake clinical placements across various hospitals during their undergraduate training. Depending on the level of study, the clinical placement lasts 30–40 weeks per academic year, and students have rotations across units with different radiological modalities and patient groups, such as geriatric patients, where more hearing‐impaired patients would be seen. However, to the best of the authors' knowledge, no study investigated their experiences in working with hearing‐impaired patients and the implications of this for radiography training, service delivery, and skills development. This study therefore aimed to investigate undergraduate Ghanaian radiography students' proficiency, experiences, confidence, and knowledge concerning communication with hearing‐impaired patients. The results of this study could provide evidence for educational institutions, radiology managers, healthcare policymakers, and other stakeholders to inform the training of radiographers, and the development and implementation of standard guidelines and policies on the management of hearing‐impaired patients. In our work, we use the term hearing‐impaired patients as an umbrella term to include the partially and completely hearing‐impaired.

## 2 Methods

### 2.1 Study Design and Site

This was a quantitative, descriptive, cross‐sectional study conducted at a public university in Ghana. The university was chosen because it runs a 4‐year undergraduate diagnostic radiography programme, and students from this programme are required to undertake periodic clinical placements across various hospitals in Ghana.

### 2.2 Study Population and Sampling Strategy

The target population for this study included all undergraduate radiography students attending the public university chosen for the study (*N* = 215). The inclusion criterion for this study was students who had completed at least one clinical placement session. First year students (*n* = 61) were excluded because they had not been on any clinical placements at the time of data collection. A convenience sampling method was used to recruit the participants. The final eligible target population comprised 154 participants (*n* = 154). Recruitment was conducted through university email and departmental social media platforms in February to April 2024. The recruitment invitation included a link to a participants' information sheet which provided information on the study and participants' rights. Students who expressed interest were then sent a consent form to confirm their participation. Once they returned the consent form, they were considered enrolled. However, the students could withdraw participation after they submitted a consent form, but before they completed the survey. Another link containing the questionnaire was sent to the participants who consented to participate in the study.

### 2.3 Data Collection Instrument and Method

A questionnaire by Nolan‐Bryant and Lockwood [9] was adopted and modified for this study. A close‐ended 16‐item structured, self‐reporting digital questionnaire (hosted on Google forms) was used to collect data for the study. The questionnaire comprised two main sections. The first section sought information on students' demographics (biological sex, age, and year of study). The second section comprised 13 questions under four sub‐headings: the ability to communicate with sign language, experience with hearing‐impaired patients, confidence in communicating effectively with hearing‐impaired patients, and knowledge on methods of communication with hearing‐impaired patients. The format of the questions included binary response questions, a three‐point attitudinal Likert scale (agree, neutral, and disagree), and multiple‐response type questions. Prior to data collection, a pilot study was conducted with a small sample of the participants (*n* = 10) to evaluate the reliability, content, and face validity of the survey, as well as the terminology, order, and grouping of questions. This was to ensure that questions were clear, aligned with the study aim, and geographically appropriate since it was adapted from a survey used in the UK. Minor amendments were made to the data collection instrument based on feedback from the pilot study. The amended questionnaire was then used in the main study. The pilot study responses were not part of the main study.

### 2.4 Data Handling and Analysis

The data was inputted into the Statistical Package for the Social Sciences (SPSS) version 26.0 for analysis. Descriptive statistics such as minimum, maximum, mean, and standard deviation were used to describe students' age, whereas frequencies and percentages were used for categorical variables (biological sex and year of study). The non‐parametric Chi‐square inferential statistics were conducted to determine statistically significant associations between variables. The Fisher's exact value was reported for the variable with two counts. A *p*‐value ≤ 0.05 was deemed statistically significant. Results were presented in tables and figures.

### 2.5 Ethics Considerations

Ethics approval for this study was obtained from the Research and Ethics Committee of the host university [REC A.4 [38] 23‐24]. Participation in this study was voluntary and anonymous. Because the survey was anonymous, the participants were only able to withdraw up to the submission of the survey. To eliminate power imbalance and prevent dual‐role conflict, recruitment was driven by a student rather than academics on the programme team.

## 3 Results

### 3.1 Demographics

Out of the 154 students invited for the study, 152 surveys were submitted, resulting in a response rate of 98.7%. The participants [males = 93 (61.2%), females = 59 (38.8%)], were aged between 17 to 32 years (mean = 21 ± 2.3 years). The distribution of the participants across the various years of study is as follows: second year (*n* = 70, 46.1%), third year (*n* = 52, 34.2%), and fourth year (*n* = 30, 19.7%). The four sub‐headings of section B of the questionnaire were used to organise the presentation of the results in sections to follow.

### 3.2 Ability to Communicate With Sign Language

Most participants (*n* = 147, 96.7%) could not communicate in sign language, whilst a few could (*n* = 5, 3.3%). The five participants that could communicate in sign language learnt this from family and friends. As shown in Table 1, the results of the Chi‐square tests indicated that there were no statistically significant associations between the students' ability to communicate using sign language and their demographics (biological sex, year of study, and age).

**TABLE 1:** The distribution of students' ability to use sign language relative to demographic variables.

Variable		Total	I can communicate using sign language		*p*
			Yes	No	
		*n* (%)	*n* (%)	*n* (%)	
Biological sex	Male	93 (61.2)	4 (4.3)	89 (95.7)	0.649
	Female	59 (38.8)	1 (1.7)	58 (98.3)	
Year of study	Year 2	70 (46.1)	0 (0.0)	70 (100.0)	0.062
	Year 3	52 (34.2)	4 (7.7)	48 (92.3)	
	Year 4	30 (19.7)	1 (3.3)	29 (96.7)	
Age (years)	17–20	67 (44.1)	2 (3.0)	65 (97.0)	0.901
	21–24	73 (48.0)	3 (4.1)	70 (95.9)	
	25–28	9 (5.9)	0 (0.0)	9 (100.0)	
	29–32	3 (2.0)	0 (0.0)	3 (100.0)	

### 3.3 Experiences Working With Hearing‐Impaired Patients Categorised as Positive or Negative

The majority of participants (*n* = 103, 67.8%) had not interacted with hearing‐impaired patients (Table 2). The results of the Chi‐square tests indicated that there was a statistically significant association between the students' experiences working with hearing‐impaired patients and their year of study (*p* = 0.017).

**TABLE 2:** Students' interaction with hearing‐impaired patients relative to demographic variables.

Variable		Total	Have you ever interacted with hearing‐impaired patients whilst on clinical placement?		*p*
			Yes	No (%)	
		*n* (%)	*n* (%)	*n* (%)	
Biological sex	Male	93 (61.2)	28 (30.1)	65 (69.9)	0.483
	Female	59 (38.8)	21 (35.6)	38 (64.4)	
Year of study	Year 2	70 (46.1)	17 (24.3)	53 (75.7)	**0.017**
	Year 3	52 (34.2)	16 (30.8)	36 (69.2)	
	Year 4	30 (19.7)	16 (53.3)	14 (46.7)	
Age (years)	17–20	67 (44.1)	19 (28.4)	48 (71.6)	0.838
	21–24	73 (48.0)	26 (35.6)	47 (64.4)	
	25–28	9 (5.9)	3 (33.3)	6 (66.7)	
	29–32	3 (2.0)	1 (33.3)	2 (66.7)	

*Note:* Bold values are used to easily identify statistically significant values.

Table 3 reports the participants overall experiences working with hearing‐impaired patients and the factors that influenced their experiences. Forty‐nine participants (32.2%) indicated that they had interacted with hearing‐impaired patients, and just over half of them (*n* = 27, 55.1%) had positive experiences. The use of body gestures (hands, lips, and mouth movements) were identified as the main contributors to their positive experiences. Whereas the lack of the following resources were identified as the main factors making participants' experiences more negative: sign language interpreters (*n* = 21, 28.4%), knowledge of sign language (*n* = 20, 27.0%), and inclusive materials (*n* = 20, 27.0%).

**TABLE 3:** Experiences of students working with hearing‐impaired patients.

Variable	Responses	Frequencies (%)
Have your experiences working with hearing‐impaired patients been positive or negative?	Positive	27 (55.1)
	Negative	22 (44.9)
Which of the following contributed to making your experience positive? (tick all that apply)	Writing	8 (13.8)
	Body gestures (hands, lips and mouth movements)	32 (55.2)
	Sign language interpreter	9 (15.5)
	Speaking loudly	9 (15.5)
Which of the following contributed to making your experience negative? (tick all that apply)	Lack of sign language interpreters	21 (28.4)
	Inability of patients to read and/or write	13 (17.6)
	Lack of knowledge of sign language	20 (27.0)
	Lack of inclusive materials (picture cards etc.)	20 (27.0)

### 3.4 Level of Confidence in Communicating With Hearing‐Impaired Patients

**FIGURE 1:**
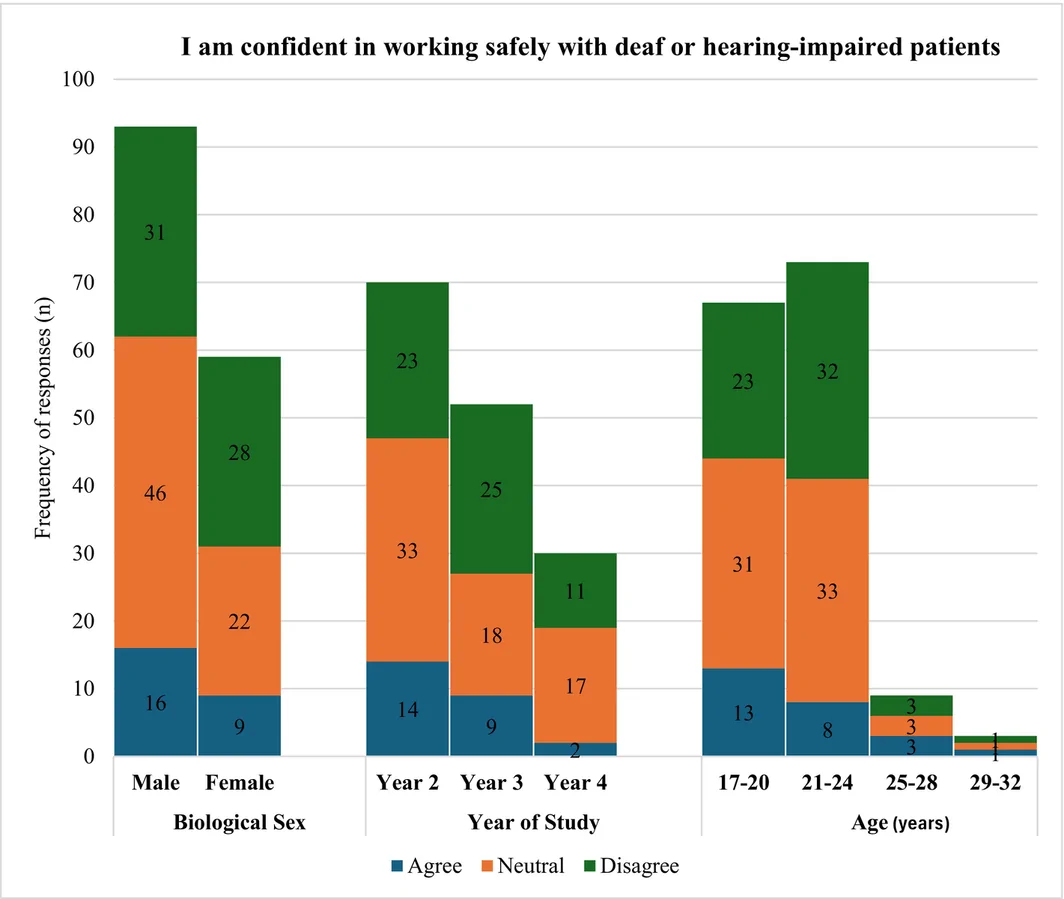
The distribution of students' confidence in working safely with hearing‐impaired patients.

Table 4 demonstrates the participants' level of confidence in communicating with hearing‐impaired patients. Most participants reported low levels of confidence in communicating with hearing‐impaired patients (*n* = 76, 50%) and obtaining informed consent from such patients (*n* = 61, 40.1%). Figure 1 presents participants' level of confidence in working safely with hearing‐impaired patients (i.e., the absence of harm to both the patient and radiographer). The majority of the participants across the three demographic variables responded in a neutral manner to the question. The results of Chi‐square tests indicated statistically significant associations between students' biological sex and their level of confidence in communicating effectively with hearing‐impaired patients (*p* = 0.043), as well as their level of confidence in gaining informed consent from hearing‐impaired patients (*p* = 0.004).

**TABLE 4:** The distribution of students' confidence in communicating effectively with, and gaining informed consent from hearing‐impaired patients.

Variable		Total	I can confidently communicate effectively with hearing‐impaired patients			*p*	I can confidently gain informed consent from patients			*p*
			Agree	Neutral	Disagree		Agree	Neutral	Disagree	
		*n* (%)	*n* (%)	*n* (%)	*n* (%)		*n* (%)	*n* (%)	*n* (%)	
Biological sex	Male	93 (61.2)	11 (11.8)	42 (45.2)	40 (43.0)	**0.043**	13 (14.0)	52 (55.9)	28 (30.1)	**0.004**
	Female	59 (38.8)	2 (3.4)	21 (35.6)	36 (61.0)		8 (13.6)	18 (30.5)	33 (55.9)	
Year of study	Year 2	70 (46.1)	6 (8.6)	34 (48.6)	30 (42.9)	0.382	11 (15.7)	35 (50.0)	24 (34.3)	0.663
	Year 3	52 (34.2)	5 (9.6)	16 (30.8)	31 (59.6)		6 (11.5)	21 (40.4)	25 (48.1)	
	Year 4	30 (19.7)	2 (6.7)	13 (43.3)	15 (50.0)		4 (13.3)	14 (46.7)	12 (40.0)	
Age (years)	17–20	67 (44.1)	3 (4.5)	32 (47.8)	32 (47.8)	0.088	9 (13.4)	29 (43.3)	29 (43.3)	0.833
	21–24	73 (48.0)	7 (9.6)	26 (35.6)	40 (54.8)		10 (13.7)	35 (47.9)	28 (38.4)	
	25–28	9 (5.9)	3 (33.3)	3 (33.3)	3 (33.3)		1 (11.1)	4 (44.4)	4 (44.4)	
	29–32	3 (2.0)	0 (0.0)	2 (66.7)	1 (33.3)		1 (33.3)	2 (66.7)	0 (0.0)	

*Note:* Bold values are used to easily identify statistically significant values.

### 3.5 Knowledge on Communication Methods for Hearing‐Impaired Patients

Table 5 indicates participants' knowledge about communication methods that can be utilised for hearing‐impaired patients. The majority of the participants (*n* = 123, 36.2%) indicated that informed consent can be gained through communicating using sign language. Most participants (*n* = 119, 29.0%) indicated that radiography departments can accommodate hearing‐impaired patients using visual alert displays (i.e., a system that uses visual cues such as flashing lights or digital signage to communicate with hearing‐impaired patients). Sign language training was also identified by the majority of participants (*n* = 132, 36.9%) as means to enhance communication between radiographers/interns and hearing‐impaired patients.

**TABLE 5:** Students' knowledge on communication methods for hearing‐impaired patients.

Variables	Responses	Frequencies (%)
Informed consent can be gained through? (tick all that apply)	Communicating using sign language	123 (36.2)
	Writing for patients to read	121 (35.6)
	Lip‐reading and demonstrative actions	96 (28.2)
How can imaging departments accommodate hearing‐impaired patients? (tick all that apply)	Availability of visual alert displays	119 (29.0)
	Availability of assistive communication devices (these are defined as specialised devices, tools, software, or equipment designed to enhance communication with hearing‐impaired patients)	113 (27.6)
	Having speech to text mobile phones	100 (24.4)
	Having a dedicated disability radiographer (i.e., a well‐trained radiographer solely responsible for providing inclusive, and high‐quality radiological services tailored to the specific needs of patients with disabilities)	78 (19.0)
Communication between radiographers/interns and hearing‐impaired patients can be improved through? (tick all that apply)	Sign language training for radiographers	132 (36.9)
	Availability of assistive communication devices	121 (33.8)
	Printing instruction for reading purposes	105 (29.3)

## 4 Discussion

The proficiency, experiences, confidence, and knowledge of 152 undergraduate diagnostic radiography students (*n* = 152) at a public university in Ghana concerning communication with hearing‐impaired patients was investigated through an online validated survey. Most of the participants were males, in their 2nd year of study, and aged between 21 and 24 years (Table 1). These demographics were expected since the 2nd year, male students between 21 and 24 years of age accounted for most of the cohort.

### 4.1 Ability to Communicate Using Sign Language

The majority of participants in our study reported not being able to communicate using sign language (Table 1). This finding is consistent with other studies which indicated that the majority of HCPs and interns cannot communicate using sign language [32, 37, 38, 39, 40]. Competency in the use of sign language is important to interact with hearing‐impaired individuals, especially in healthcare settings, because sign language is the dominant form of communication used by hearing‐impaired patients [41]. The inability of the students in our study to communicate with hearing‐impaired patients via sign language could lead to violation of patients' rights, safety incidents, and reduced quality of care [38]. These adverse outcomes could lead to mistrust, fear, and frustration with HCPs, interns, and the healthcare system [42].

Although a few students indicated that they could communicate using sign language (Table 1), they had their training through family and friends—an informal training method. This finding is similar to a study by Alamro et al. [29] where HCPs who could communicate using sign language learnt the technique through self‐study. Although informal learning could have some advantages, it is mostly less effective and incomprehensive relative to formal learning [43]. Additionally, informal learning methods mostly have no standardised learning practices, knowledge accumulation, and assessment methodologies [44, 45, 46, 47]. None of the participants in our study had formal training in the use of sign language. This therefore demonstrates the need for the incorporation of sign language training into the undergraduate curricula for the training of diagnostic radiography students to improve their competence, which could facilitate better patient outcomes. The content of such training must take into consideration the self‐efficacy of the students and cover key vocabulary, phrases, and common procedures [48]. It must ensure that students develop both their ability to use and understand sign language when used by others.

However, to improve communication with hearing‐impaired patients, supplementation of sign language with visual aids is helpful, as not all hearing‐impaired patients use formal sign language or have had access to formal training [49]. Also, to enhance the effectiveness of the use of sign language between radiographers/interns and hearing‐impaired patients, there is the need for cultural competency development to narrow the cultural gaps between these two groups [50, 51]. Cultural competency could also aid in improving the self‐efficacy of HCPs, dispel misconceptions, deepen awareness, and improve communication with hearing‐impaired patients [48, 52, 53]. Despite the benefits of teaching and learning sign language, practical challenges may include the lack of qualified sign language teachers, a shortage of learning materials, and a lack of funding especially in low‐resource countries such as Ghana.

### 4.2 Students' Experiences Working With Hearing‐Impaired Patients

Most participants in our study had no prior experience interacting with hearing‐impaired patients (Table 2). This finding is consistent with other studies where most healthcare students had not interacted with hearing‐impaired patients [23, 40, 54, 55]. In contrast, a study by Nolan‐Bryant and Lockwood [9] involving UK radiography students indicated that over 80% of the students reported at least one interaction with hearing‐impaired patients during clinical placements. In our study, the limited interaction of students with hearing‐impaired patients could be attributed to two main reasons. First, as stated earlier, only 2% of the Ghanaian population have hearing impairment; therefore, it is less likely that students will have interacted with such patients. Secondly, Obosu et al. [25] indicated that the few Ghanaians with hearing impairment barely seek healthcare. This could be due to communication barriers, marginalisation, past negative experiences, and stigmatisation [52, 53, 56]. According to Duorinaah et al. [49], professional stigma—the attitude of HCPs towards patients with disabilities such as the hearing‐impaired—is very common in Ghana. Additionally, the hearing‐impaired community faces difficulty accessing health information due to the lack of health information and education materials tailored to them, which can negatively impact their health‐seeking behaviours [57]. This emphasises the need for formal education, as previously discussed, to mitigate these negative perceptions.

Also, the results of our study showed a statistically significant association between students' year of study and their interaction with hearing‐impaired patients (*p* = 0.017), indicating that participants with more years of study are likely to have more interactions with hearing‐impaired patients because they have completed more clinical placements (Table 2). Literature highlights that students' low interaction with hearing‐impaired patients could lead to the reinforcement of negative attitudes and behaviours because there is evidence to show that the more students interact with hearing‐impaired patients, the more likely there would be changes in their perceptions, behaviours, and attitudes [48].

In our study, the majority of participants who had interacted with hearing‐impaired patients reported positive experiences (Table 3). The main factor contributing to these positive experiences was the use of body gestures (hands, lips, and mouth movements) (Table 3). This finding is consistent with a study by Nolan‐Bryant and Lockwood [9] which indicated that the majority of the students in their study had positive experiences in their interactions with hearing‐impaired patients. The clinical implication of students having positive interaction(s) with hearing‐impaired patients is that these students develop increased confidence and clinical morale, reduced burnout, and improved future interactions with hearing‐impaired patients [58, 59]. Conversely, students who reported negative experiences (Table 3) indicated the absence of sign language interpreters as a major contributing factor. This phenomenon is common in Ghana because structures to train sign language interpreters are not well established or standardised with relatively few individuals showing interest in this career [56]. Additionally, the absence of sign language interpreters could be attributed to hearing‐impaired patients' reservations about using them due to concerns about privacy and confidentiality [49]. Negative experiences with hearing‐impaired patients could lead to HCPs and interns being reluctant to attend to the hearing‐impaired. This could contribute to poor health‐seeking behaviour and negative health outcomes for such patients. Negative experiences with hearing‐impaired patients could also reinforce negative socio‐cultural stereotypes. This is pertinent in Ghana where hearing disorders are regarded as a bad omen and/or a curse from ancestral gods, with affected individuals subject to stigma and social ostracisation [53, 56].

Our study findings underscore the need to educate undergraduate diagnostic radiography students on best practice strategies of communicating with patients with a variety of hearing impairments so as to ensure person‐centred care [60]. Moreover, students need to be socialised and supported through education and training to use their scope of practice to guide their professional practice and interactions with patients so as to mitigate the negative impact of societal beliefs and stigma associated with hearing impairment [61].

### 4.3 Students' Level of Confidence in Communicating With Hearing‐Impaired Patients

Most participants in our study indicated low confidence communicating with hearing‐impaired patients (Table 4), aligning with the results of Nolan‐Bryant and Lockwood [9], though confidence levels in our cohort were notably lower. Additionally, male students in our study reported higher confidence than females (*p* = 0.043), while the UK study found no statistically significant sex‐based differences. Students with low levels of confidence in communicating effectively with hearing‐impaired patients could have negative implications for healthcare delivery because such students are likely to avoid interactions with hearing‐impaired patients [62]. As a result, these patients may be deprived of access to quality healthcare, thereby compromising the sustainable development goal three (SDG 3) which demands healthcare for all patients [63].

Few students in this study (Figure 1) were confident in working safely with hearing‐impaired patients, and in gaining informed consent from hearing‐impaired patients (Table 4). These findings are inconsistent with a study by Nolan‐Bryant and Lockwood [9] which indicated that the majority of radiography students in the UK were confident in gaining informed consent and working safely with hearing‐impaired patients. The reason for this inconsistency may be the large number of participants in our study who have not yet had any interaction with hearing‐impaired patients during clinical placement. Low levels of confidence in explaining the necessary information needed for patients to give informed consent could lead to poor communication of information and eventually confusion on the patients' part, which could limit informed decision‐making [35, 64]. Specific education in communicating to obtain informed consent from hearing‐impaired patients will be useful in this regard. Similarly, low levels of confidence in working safely with hearing‐impaired patients could lead to errors during radiography procedures [65]. This may cause harm to both patients and radiography staff, could result in medico‐legal suits, and derail trust in the healthcare delivery system [66].

### 4.4 Students' Knowledge on Communication Methods for Hearing‐Impaired Patients

Most of the participants demonstrated a high level of knowledge on communication methods for hearing‐impaired patients (Table 5). The majority of the participants indicated that informed consent from hearing‐impaired patients can be gained using sign language. This finding is consistent with other studies by Foltz and Shank [67] and Hommes et al. [68] which found that effective communication with hearing‐impaired patients is best achieved through the use of sign language. Also, other communication methods that can be used in obtaining informed consent, as reported by the participants in our study, included writing for patients to read and lip‐reading (Table 5). However, the effectiveness of writing depends on the patients' literacy level, and most patients with hearing disorders are not fluent in written English, as found in two African studies conducted in South Africa and Kenya [31, 69]. This could be more severe in Ghana, where cultural, social, economic, and systemic barriers prevent persons with disabilities from accessing formal specialist education, thereby limiting their proficiency in written English [70, 71]. Although hearing‐impaired individuals may develop lip‐reading skills, experts estimate that over 70% of spoken English is inaccurately interpreted, making lip‐reading a poor form of primary communication [68]. Ogwudu & Akpaniwo [36] further added that accents and face coverings can make it difficult for the few hearing‐impaired individuals who rely on lip‐reading to communicate effectively. To enhance effective communication, there is a need for radiography students to know the communication preferences of hearing‐impaired patients and develop specific strategies to meet these demands [57]. The majority of participants in our study also indicated that radiography departments can accommodate hearing‐impaired patients using visual alert displays (Table 5). This position is supported by Hong et al. [72], who indicated that hearing‐impaired individuals largely depend on visual aids for communication and interactions with their environment.

A further teaching and learning strategy that could be considered to enable students to more appropriately interact and communicate with hearing‐impaired patients is simulation. Literature highlights that simulation not only facilitates developing effective communication skills but also other behaviours and attitudes key to person‐centred care such as empathy and understanding patient preferences. Additionally, it aids in the development of a deeper understanding of the importance of radiographer position and body language relative to the patient and speech speed which may influence patients' lip‐reading ability and understanding of the message trying to be conveyed [73, 74]. In using simulation‐based learning strategies, students can be better prepared to engage appropriately with hearing‐impaired patients in the clinical setting. Another strategy that could be considered is patient stories to cultivate students' interpersonal skills required to facilitate effective communication with hearing‐impaired patients. Moreover, students develop critical reflective skills to constantly reflect on their own practices and internalise best practice behaviours, attitudes, and professional knowledge so as to provide more person‐centred care practices [75, 76].

### 4.5 Limitations

This is a single‐centre study, and the findings are not generalisable to all Ghana‐based radiography student populations. Students' personal views towards hearing‐impaired patients, and the self‐reporting nature of the questionnaire may also have influenced their responses.

### 4.6 Recommendations

This study highlights the need for educational reform to include content in the undergraduate diagnostic radiography curriculum to enable students develop appropriate knowledge and confidence to interact with and care for hearing‐impaired patients, as well as equip students with basic sign language skills. Larger‐scale multicentred studies on this topic can be conducted to obtain more generalisable findings. Furthermore, systematic reviews and document analyses can be conducted to synthesise evidence on best practices regarding learning and teaching strategies to address the curriculum gap identified.

## 5 Conclusions

A survey of Ghanaian undergraduate diagnostic radiography students' proficiency, experiences, confidence, and knowledge concerning communication with hearing‐impaired patients was conducted at one university. Sign language proficiency was minimal among the participants. The majority of participants had no prior interactions with hearing‐impaired patients during clinical placement. Participants also demonstrated some knowledge about effective communication practices in the context of working with hearing‐impaired patients, but their confidence is limited.

## Funding

The authors have no funding to report.

## Ethics Statement

Ethics approval for this study was obtained from the Research and Ethics committee of the University of Health and Allied Sciences (UHAS), Ho, Ghana [UHAS‐REC A.4 [38] 23‐24]. The study conformed with the Declaration of Helsinki and the Belmont Report.

## Consent

Participation in this study was voluntary and anonymous. Participants were informed that they had the right to withdraw from the study at any time without providing a reason, and that their non‐participation would not yield any repercussions.

## Conflicts of Interest

The authors declare no conflicts of interest.

## Data Availability

The data used to support this study are available within the article. Additional data can be made available by the corresponding author upon request.
